# Social isolation during the COVID-19 pandemic in Spain: a population study

**DOI:** 10.1038/s41598-022-16628-y

**Published:** 2022-07-22

**Authors:** Marina Martínez-Garcia, Emilio Sansano-Sansano, Andrea Castillo-Hornero, Ruben Femenia, Kristof Roomp, Nuria Oliver

**Affiliations:** 1 Universitat Jaume I, Castelló, 12071 Spain; 2 Microsoft Corporation, Washington, USA; 3 ELLIS Unit Alicante Foundation, Alicante, Spain

**Keywords:** Public health, Population screening

## Abstract

Since March of 2020, billions of people worldwide have been asked to limit their social contacts in an effort to contain the spread of the SARS-CoV-2 virus. However, little research has been carried out to date on the impact of such social distancing measures on the social isolation levels of the population. In this paper, we study the impact of the pandemic on the social isolation of the Spanish population, by means of 32,359 answers to a citizen survey collected over a period of 7 months. We uncover (1) a significant increase in the prevalence of social isolation in the population, reaching almost 26%; (2) gender and age differences, with the largest prevalence of isolation among middle-aged individuals; (3) a strong relationship between economic impact and social isolation; and (4) differences in social isolation, depending on the number of COVID-19 protection measures and on the perception of coronavirus infection risk by our participants. Our research sheds quantitative light on the sociological impact of the pandemic, and enables us to identify key factors in the interplay between the deployment of non-pharmaceutical interventions to contain the spread of an infectious disease and a population’s levels of social isolation.

## Introduction

Social isolation is defined as “the relative absence of social relationships”^1^. It is increasingly recognised as an important public health issue^2^. In fact, numerous studies have found that social isolation, particularly among older adults, is a risk factor associated with premature death, mainly due to cardiovascular or mental health problems^3–6^, with an effect similar to that of obesity, a sedentary lifestyle, substance abuse and cigarette smoking^7^. This negative association with health is found even when controlling for other variables, such as socioeconomic factors, perceived loneliness or life habits^7,8^. In recent work by Holt-Lunstad and Steptoe^9^, the authors discuss three elements of social relationships (namely function, structure and quality) and their impact on an individual’s health. While they claim that “none of these three elements alone will adequately capture the full scope of social influence on health”, they hypothesise that social isolation “may be a critical component because a weak structural foundation may limit the potential of other social connection factors to have downstream effects on health”.

For this reason, developing instruments to assess the levels of social isolation in a population is of paramount importance given that social engagement—i.e., the lack of social isolation—is a key pillar for good health. Different instruments have been developed in the literature to quantify the levels of social isolation in individuals, such as the ENRICHD Social Support Instrument, the Berkman-Syme Social Network Index and the Duke Social Support Indices^10,11^. The Lubben Social Network Scale (LSNS) is one such instrument. The original version of the LSNS scale consists of 10 items and was originally developed by Lubben in 1988 to assess the social isolation in older adults^12^. Recently, a six-item version of the LSNS, called the LSNS-6, was developed by Lubben and Gironda^13–15^ and has been validated as a reliable instrument to assess the levels of social isolation both in older^13,16–18^ and younger adults^19^.

A related concept to social isolation is that of social capital, which refers to the institutions, relationships and norms that shape the quality and quantity of a society’s social interactions^20^. Social capital has both an individual and an aggregate component^21–23^. Generally, social capital consists of three dimensions: structural, relational and cognitive. The LSNS-6 measures the relational and structural elements of the social network of an individual by assessing both the social support and the social network aspects, which are part of a person’s individual social capital^24,25^.

Older adults have been reported to be the most vulnerable demographic group to social isolation, mainly due to their reduced physical mobility and general decline in health status, life transitions associated with age (e.g. retirement, loss of a partner, relatives and friends) and ageism in society^11,26–28^. Beyond the elder population, there are few studies that have assessed the prevalence of social isolation in the general population prior to the COVID-19 pandemic^29,30^.

However, the pandemic has brought social isolation to the public eye, not only in the context of older adults, but for all age groups, because of the impact that confinement and social distancing measures have on everyone’s social support structures^31^. During the pandemic, billions of people worldwide have been asked to limit their social contacts by physical distancing, home confinements and the temporary closure of many social activities—such as restaurants, bars, gyms, workplaces, soccer stadiums, museums, cinemas and theatres—in an effort to contain the spread of the SARS-CoV-2 virus.

Despite the importance of social isolation in this context, there are few population studies published to date that have analysed the impact of the COVID-19 pandemic on a population’s levels of social isolation^32–34^. One of the most relevant pieces of previous work is by O’Sullivan et al.^35^ where they studied the impact of the pandemic on the social isolation of the general population in 101 different countries, mainly from the United States (40%) and the UK (21%). The authors collected the answers to the LSNS-6 survey from 14,302 participants aged over 18 years old (M=53 years, SD=17.6) before and during the coronavirus pandemic. They found a 13% increase in the prevalence of social isolation during the pandemic. The most important factors associated with social isolation—both before and during the coronavirus pandemic—are a lack of financial resources to meet the participants’ needs, their self-rated physical and mental health, living alone not by choice, poor sleep, low physical activity and alcohol consumption. The most cross-cutting aspects correlated with social isolation were economic and mental health factors. During the pandemic, rural residents were found to be significantly at larger risk of social isolation when compared to city dwellers.

In this paper we focus on studying the levels of social isolation in the Spanish population during the COVID-19 pandemic. Spain was one of the most affected countries in the early phases of the pandemic during the spring of 2020. In an effort to contain the spread of the virus, the government implemented an extremely restrictive lockdown that brought the country to a halt for several weeks, with tremendous economic and social implications. Spanish culture is highly social, with low levels of individualism, where families are a fundamental pillar in most people’s lives^36–39^. People in Spain tend to handle their personal problems through their family, relying on relatives for support when facing difficult situations, such as a pandemic.

In order to measure social isolation, we use the LSNS-6 instrument, delivered as part of an online, anonymous survey called the COVID19ImpactSurvey^40^, with over 720,000 answers collected since March of 2020. The answers to the survey have been previously analysed for research purposes and extensively used by public authorities, the media and citizens in Spain to measure the impact of the COVID-19 pandemic on people’s behaviours, perceptions and lives^41,42^. While the original survey did not include any questions to assess the levels of social isolation of the respondents, on June 6th 2021, the LSNS-6 questions were added to the survey. We analyse a sample of 32,359 answers collected in Spain for the time period between June 6th, 2021 and December 16th, 2021.

In our analysis, we aim to answer four research questions on the interplay between the levels of social isolation in the Spanish population, the economic and psychological impact of the COVID-19 pandemic and the self-reported behaviour:

**RQ1**: What is the distribution and the temporal evolution of the levels of social isolation in the Spanish population during six months of the COVID-19 pandemic?
**RQ2**: What is the relationship between social isolation and the economic impact of the pandemic on individuals?
**RQ3**: What is the relationship between social isolation and the psychological impact of the pandemic on individuals?
**RQ4**: Are there differences in the levels of social isolation between those with low perceptions of infection risk associated with common daily activities and those who do not adopt any COVID-19 protective measures when compared to the rest of the sample?

The paper is structured as follows: in the following section, we present the Results of analysing the survey answers to address the four previously stated research questions. Next, we Discuss the results and draw key conclusions from our research. Finally, we describe our “Materials and methods”.

## Results

We analyse 32,359 answers to the COVID19ImpactSurvey, which two of the authors designed and launched on March 28th, 2020. Our results are based on analysing a subset of 17 questions of this survey as per Table S3. We focus on the answers collected between June 6th, 2021 and the 16th of December, 2021 in Spain (N = 32,359). Respondents were required to be at least 18 years old. We refer the interested reader to^40^ for a detailed description of the survey and its methodology.

The gender and age distributions of the collected sample are not proportional to those of the general population of Spain, as shown in Table S5. Thus, we follow the methodology described in Oliver et al.^40^ and re-weigh the answers, such that the resulting gender and age distributions match the official statistics in Spain in 2020.

All answers are categorical or binary, thus we report the percentage of participants who selected each response and compute the 95% confidence intervals (CI) through the margin of error. All tests of significance have been done by means of t-test or ANOVA as appropriate and post-hoc comparisons have been performed using Tukey tests.

### RQ1: distribution and temporal evolution of social isolation

#### Sample characteristics

Complete LSNS-6 scores were available for 27,898 participants 51.49% were women and 48.51% were men in the re-weighted sample. The mean age of the study sample was 42.45 years. The mean LSNS-6 score of the study population was $\mu _{LSNS-6} = 15.23$ ($\hbox {SD} = 0.043$). The average LSNS-6 scores were 7.66 ($\hbox {SD}=0.041$) and 17.89 ($\hbox {SD} =0.036$) for socially isolated ($\mu _{LSNS-6}<12$) and socially integrated ($\mu _{LSNS-6}\ge 12$) individuals, respectively.

#### Prevalence of social isolation

The prevalence of social isolation was $prev_{iso} = 25.98\%$ ($\hbox {SD}=0.003$) across all ages in the sample. Social isolation was slightly more prevalent in men when compared to women (men: $prev_{iso} = 26.53\%$, women: $prev_{iso} = 25.47\%$), yet we did not identify a statistically significant difference between genders. Conversely, the LSNS-6 average scores among men were larger than among women (men: $\mu _{LSNS-6} = 15.29$, $\hbox {SD}=0.074$; women: $\mu _{LSNS-6} = 15.17$, $\hbox {SD}=0.047$).

**Figure 1:**
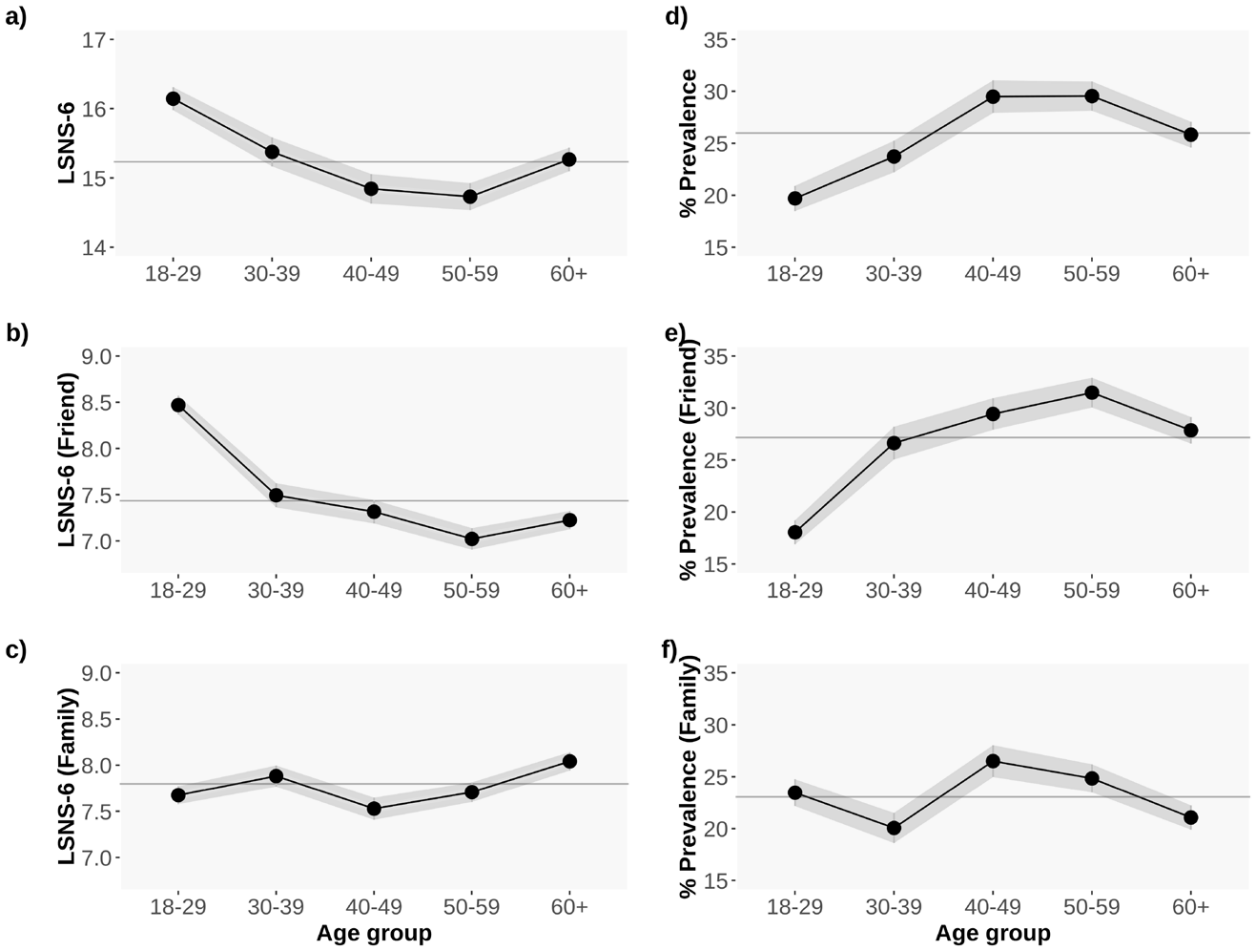
Average LSNS-6 (**a**), LSNS-6 Friends (**b**) and LSNS-6 Family (**c**) scores per age group. The larger the LSNS-6 score, the lower the levels of social isolation; Proportion of non isolated individuals per age group, defining isolation as a LSNS-6 score $< 12$ (**d**), a LSNS-6 Friend score $< 6$ (**e**), and a LSNS-6-Family score $< 6$ (**f**). The greyed area shows the 95% CI. The grey continuous line corresponds to the overall average LSNS-6 score for all the age groups: $\mu _{LSNS-6} = 15.23$, $SD = 0.043$; $prev_{iso} = 25.98\%$, $SD = 0.003$; $\mu _{LSNS-6\,fri}=7.43$, $SD = 0.026$; $\mu _{LSNS-6 \,fam}=7.80$, $SD = 0.025$; $prev_{iso\,fri}=27.16\%$; $prev_{iso\,fam}=23.04\%$.

The demographic group with the largest average LSNS-6 scores is the youth (18–29 years old), followed by the elderly (aged 60+ years old) and those aged 30 to 39 years old. Interestingly, the middle-aged (40–59 years old) group exhibits the lowest LSNS-6 values, yielding an “U”-shaped curve of LSNS-6 scores, as shown in Fig. 1a and d. In our data data, 29.54% of the individuals aged 50–59 years old report being socially isolated, a figure significantly larger than what has been previously presented in the literature prior to the pandemic^30^.

In order to shed light on the contributions of friends vs family relationships in the overall LSNS-6 score, Fig. 1 b, c, e and f show the average LSNS-6 Friend and Family scores and the average prevalence of social isolation per age group. As observed in the Figure, the LSNS-6 scores in the youth (18–29 years old) are strongly reliant on the Friends component whereas the Family component is more important in the elderly (60+ years old): $\mu _{LSNS-6 \,fam}$: 7.67 in the youth vs 8.04 in the elderly; $\mu _{LSNS-6 \,fri}$: 8.47 in the youth vs 7.22 in the elderly.

**Figure 2:**
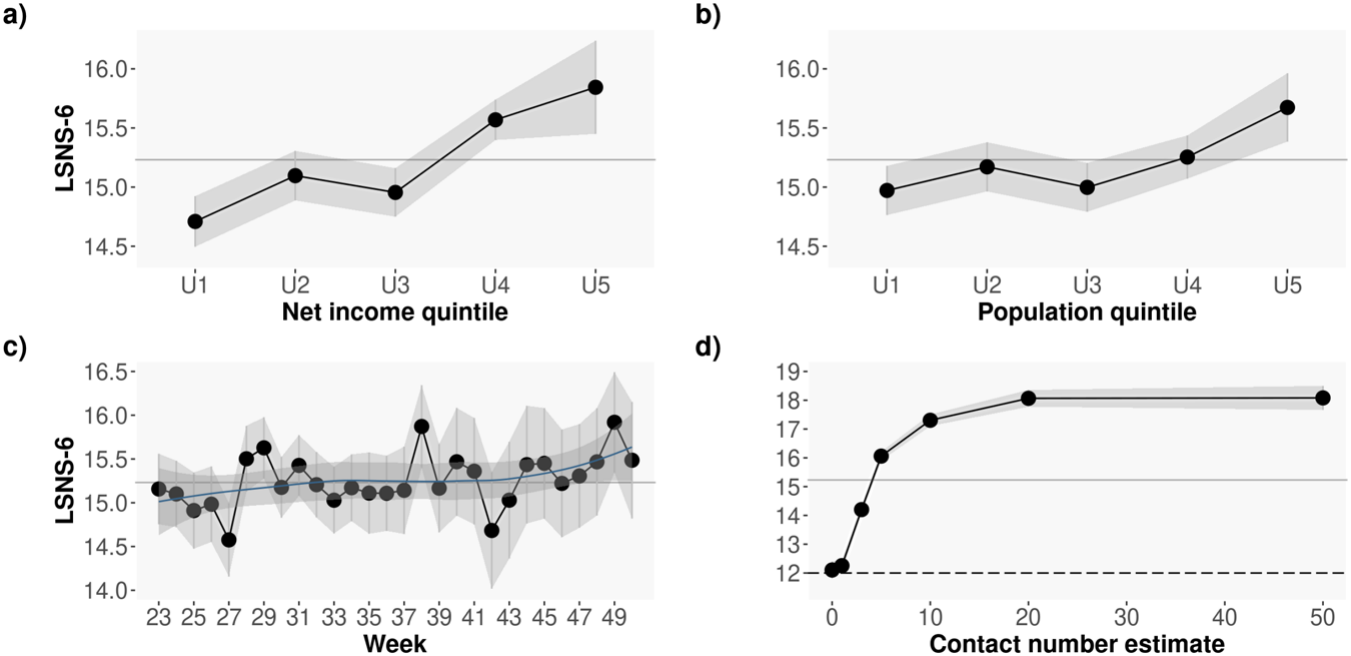
Average LSNS-6 score by income (**a**) and population (**b**) in thousands, both obtained from the 2020 Spanish official statistics (INE) data according to the participants’ reported postal code. The larger the income and the larger the population, the lower the levels of social isolation; (**c**) Average LSNS-6 score by week of the year: from June 6th to December 16th, 2021; (**d**) Average LSNS-6 score by the number of close contacts in the last 7 days. Note how the larger the number of close contacts, the larger the LSNS-6 scores and hence the lower the levels of social isolation. The greyed area shows the 95% CI. The grey continuous line correspond to the overall average LSNS-6 for the entire sample: $\mu _{LSNS-6} = 15.23$, $SD = 0.043$.

The temporal evolution of the average LSNS-6 scores over time is shown in Fig. 2a. As depicted in the Figure, the average values are generally stable over time except for a notable decrease in the LSNS-6 scores collected during week 27 (5th to 11th of July, 2021) and an increase in the LSNS-6 scores corresponding to week 38 (20th–26th of September, 2021), which might be due to the return to work and school after the summer holidays. A LOESS curve fit reveals a trend of larger average LSNS-6 scores starting in week 43, which might be reflective of the return to the “new normality” and the emergence of *pandemic fatigue* in the population^41^.

#### Associations between social isolation and socio-demographic and economic factors

Previous work has identified significant correlations between social isolation, income and population density^5,8^. In our data, we observe a similar pattern to that reported in the literature: Fig. 2b and c depict the average LSNS-6 scores for all the participants living in postal codes grouped by their associated income (Fig. 2b) and population (Fig. 2c) as reported by the Spanish National Institute of Statistics (data for 2020). As seen in the Figure, the larger the levels of isolation (i.e. the lower the LSNS-6 score), the lower the income and the lower the population: those living in poorer areas and those living in smaller municipalities exhibit significantly lower LSNS-6 scores than those living in more affluent or/and populated regions.

A logistic regression analysis confirmed associations of age, number of people living in the household, average income and population of the respondents’ zip code with social isolation, as depicted in Table 1. In the Table, the income is divided in three bins: low [12,201–22,064 €/year]; medium [22,065–25,414 €/year]; and high [25,415–60,263 €/year], and so is the population: low [119–44,320 inhabitants]; medium [44,321–312,004 inhabitants]; and high [312,005–5,226,965 inhabitants].

**Table 1:** Logistic regression analysis of social isolation with age, gender, number of people in the home, income, population, economic and psychological impact as independent variables. The asterisks denote statistical significance of the coefficient in the logistic model,* p*-values $$0.001<^{***}, 0.01<^{**}, 0.05<^{*}$$.

	Odds ratio	Int low	Int sup
**Age Group: Basal value 18–9**			
30–39$$^{***}$$	1.29	1.12	1.48
40–49$$^{***}$$	1.83	1.61	2.08
50–59$$^{***}$$	1.89	1.67	2.14
60+$$^{***}$$	1.59	1.39	1.81
**Gender: Basal value Female**			
Male	1.07	1.00	1.16
**Number people household: Basal value 1**			
2$$^{***}$$	0.71	0.63	0.80
3$$^{***}$$	0.73	0.64	0.83
4+$$^{***}$$	0.65	0.57	0.74
**Income: Basal value Low**			
Medium	1.02	0.93	1.12
High$$^{***}$$	0.77	0.68	0.86
**Population: Basal value Low**			
Medium$$^{**}$$	0.86	0.78	0.94
High	1.00	0.90	1.11
**Economic impact: Basal value None**			
Positive$^{*}$	0.89	0.79	1.00
Mild$$^{***}$$	1.18	1.08	1.30
Severe$$^{***}$$	1.90	1.58	2.27
**Psychological impact: Basal value None**			
Mild$$^{**}$$	0.84	0.74	0.95
Severe$$^{***}$$	1.39	1.27	1.51

#### Relationship between the contact number estimate and LSNS-6 scores

Question 9 in the survey (see Table S3) asks participants to estimate the number of different people from outside the home they have had a close contact with in the past week. This question is meant to assess the intensity of social relationships in the survey respondents. Figure 2d shows the relationship between the reported number of close contacts and the LSNS-6 scores of the participants in our sample. As seen in the Figure, the larger the number of close contacts, the higher the LSNS-6 scores, i.e. the lower the probability of social isolation.

### RQ2: social isolation and economic impact of the pandemic

We address RQ2 by analysing the answers to questions Q20 and Q22 in the survey (see Tables S3 and S4), as described next.

**Figure 3:**
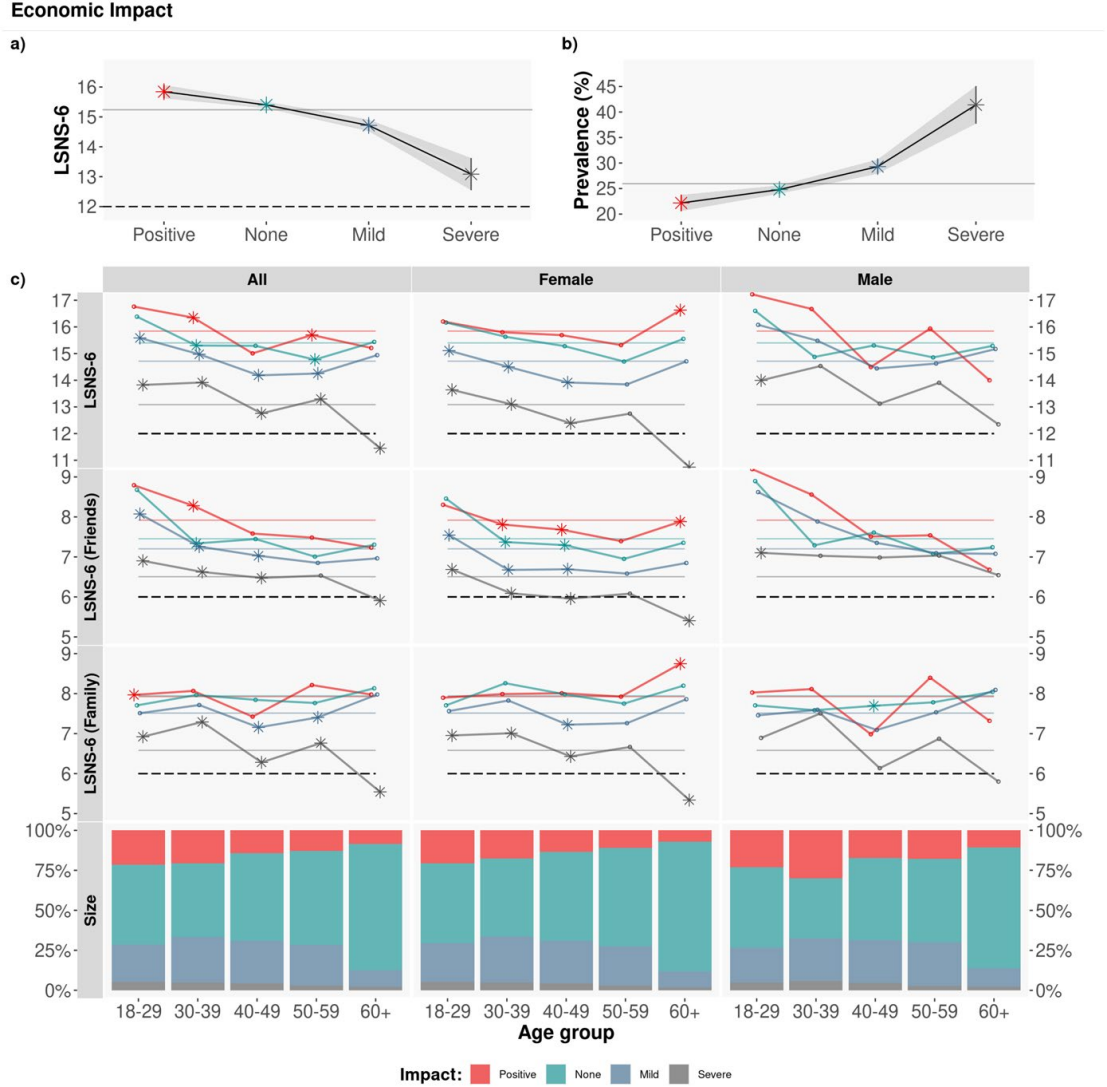
Economic impact. Average LSNS-6 score by age group and gender. The coloured dashed lines correspond to the Overall/Friend/Family average LSNS-6 score per economic impact (Positive/None/Mild/Severe): $\mu _{LSNS-6}$: [15.84, 15.40,14.71, 13.09]; $\mu _{LSNS-6\,fam}$: [7.92, 7.94, 7.51, 6.58]; $\mu _{LSNS-6\,fri}$: [7.92, 7.45, 7.20, 6.51]. To test the statistical significance of the differences, we applied a Tukey post-hoc test after obtaining a significant difference via an ANOVA test. Asterisks denote statistically significant differences with the rest of groups with $\alpha =0.05$. Note how the more severe the economic impact, the lower the LSNS-6 scores and hence the larger the levels of social isolation.

Figure 3 shows the results of the average LSNS-6 scores per age group, gender and severity of the economic impact of the pandemic. As seen in the Figure, those who report severe economic impact have significantly lower LSNS-6 scores when compared to the rest (ANOVA, * p* $<10^{-16}$, all with post-hoc Tukey* p* $<10^{-6}$). Over 40% of participants with severe economic impact report being socially isolated. This difference in the LSNS-6 scores is particularly significant for female respondents when compared to male respondents of the same age with severe economic impact, as illustrated in Fig. 3, bottom. In terms of friends vs family, the LSNS-6 score related to friends is the largest contributor to this very clear gender difference. Regarding age, those aged 60+ with severe economic impact have the lowest LSNS-6 scores of the entire sample: 50.56% of participants in this group report being socially isolated.

Table 1 shows the results of a logistic regression analysis of social isolation with age, gender, number of people in the home, income, population, economic and psychological impact as independent variables. All levels of psychological and economic impact are positively associated with social isolation: the larger the psychological and/or economic impact, the larger the levels of social isolation.

In addition to the type of economic impact, we analysed the relationship between social isolation and having received any kind of government economic assistance in the past month (Q22 in the survey).

**Figure 4:**
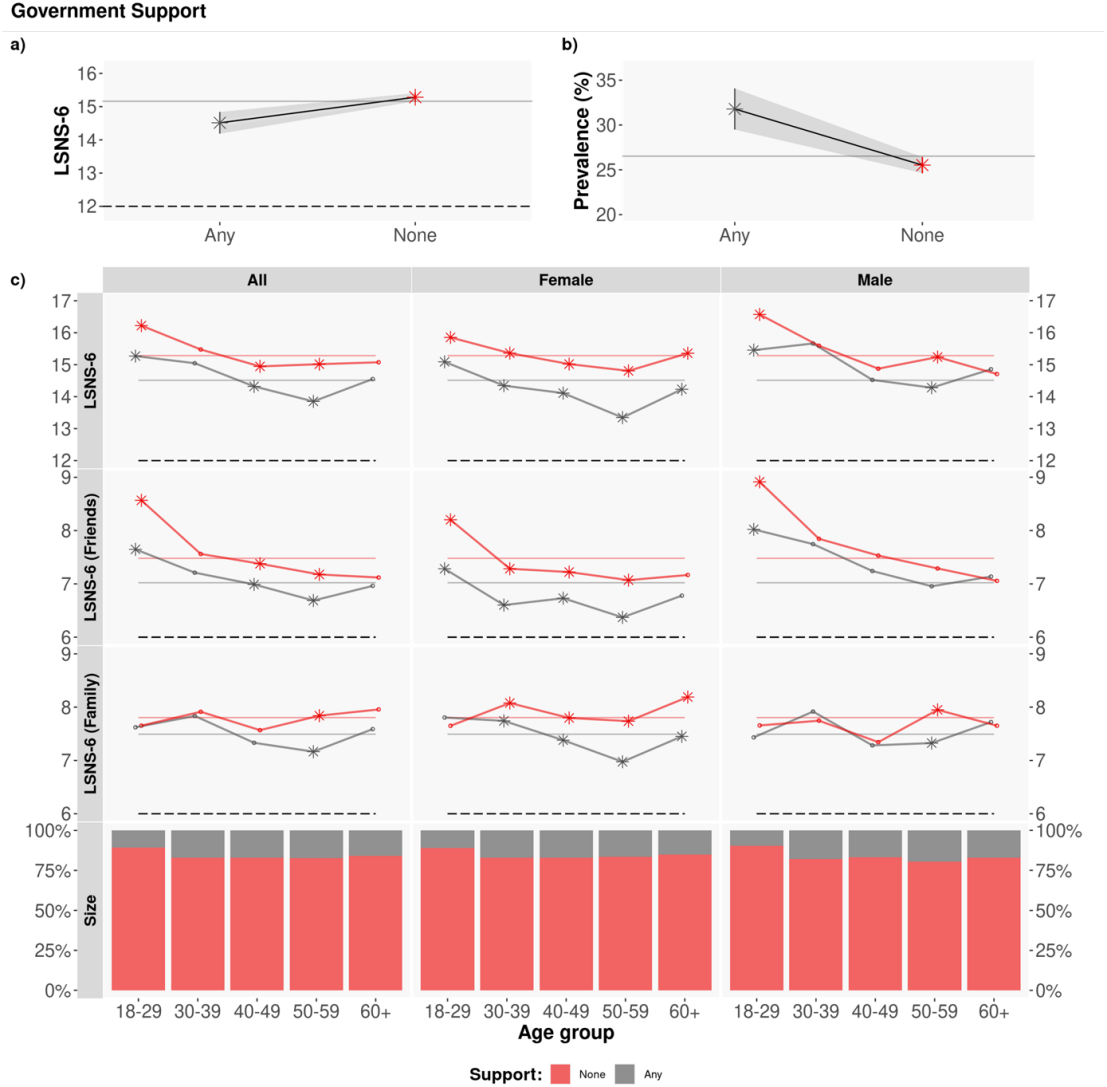
Government support. Average LSNS-6 score by age group and gender. The coloured dashed lines correspond to the Overall/Friend/Family average LSNS-6 score per government economic assistance (Any/None): $\mu _{LSNS-6}$: [14.50, 15.34]; $\mu _{LSNS-6\,fam}$: [7.47,7.77]; $\mu _{LSNS-6\,fri}$: [7.03, 7.55]. The asterisks denote statistically significant differences, via a t-test with $\alpha =0.05$. The LSNS-6 scores of those receiving government support (gray lines) are significantly lower than the scores of those who do not receive any kind of government support (red lines).

Figure 4 depicts the LSNS-6 scores for those who report receiving government support (red lines) vs those not receiving any type of support by the government (grey lines) per age group and gender. As shown in the Figure, the average LSNS-6 scores of those receiving government support are significantly lower than the scores of those who do not report receiving any kind of support ($\mu _{LSNS-6}$: 14.50 vs. 15.34 for those receiving government support vs not, t-test $p<10^{-9}$ with a difference in means of 0.836).

The largest differences are found in the friend component of the LSNS-6 score, particularly in female respondents.

In terms of age, the lowest average LSNS-6 scores correspond to those who receive government support and are 50–59 years old, where 35.35% of individuals in this group report being socially isolated.

### RQ3: social isolation and the psychological impact of the pandemic

We address RQ3 by analysing the answers to question Q31 in the survey (see Tables S3 and S4).

**Figure 5:**
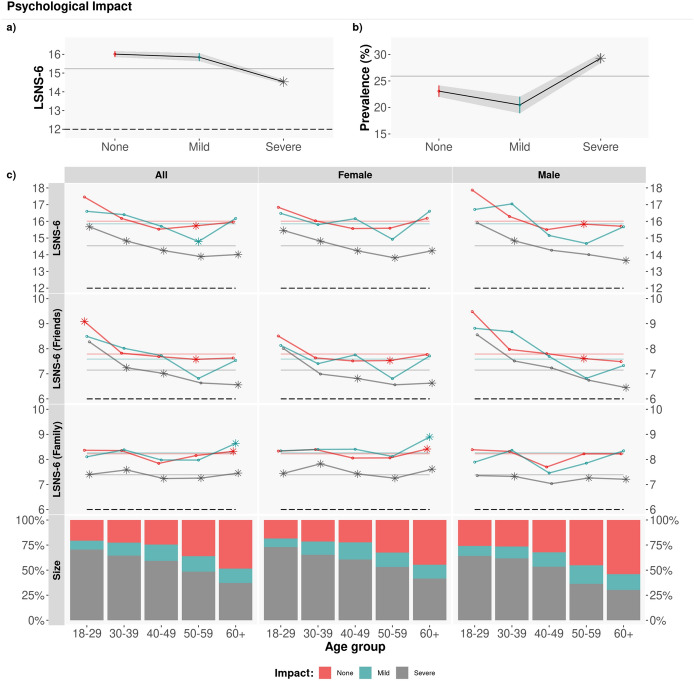
Psychological impact. Average LSNS-6 score by age group. The coloured dashed lines correspond to the average overall/friend/family LSNS-6 score depending on the psychological impact (None/Mild/Severe): $\mu _{LSNS-6}$: [16.01, 15.85, 14.54]; $\mu _{LSNS-6\,fam}$: [8.23, 8.26, 7.39]; $\mu _{LSNS-6\,fri}$: [7.79, 7.59, 7.15]. To test the statistical significance of the differences, we applied a Tukey post-hoc test after obtaining a significant difference via an ANOVA test. Asterisks denote statistically significant differences with the rest of groups with $\alpha =0.05$. The average LSNS-6 scores of those with severe psychological impact are significantly lower than the rest.

Figure 5 shows the average LSNS-6 scores per age group, gender and severity of the self-reported psychological impact of the pandemic ($\mu _{LSNS-6}$: None/Mild/Severe: [16.01, 15.85, 14.54]). Similarly to economic impact, those who report severe psychological impact have significantly lower LSNS-6 scores than the rest of the respondents. Interestingly, we do not identify significant differences by gender or by type of social support (friends or family) among those who report severe psychological impact. The lowest average LSNS-6 scores correspond to those aged 50 to 59 years old: 29.54% of respondents in this age group report being socially isolated, and 34.90% for those who report severe psychological impact.

A logistic regression analysis shown in Table 1 confirmed associations between psychological impact and social isolation in our sample, yet milder than the associations found with economic impact.

### RQ4: social Isolation, Behaviours and Perceptions related to COVID-19 measures

The fourth research question focuses on the relationship between social isolation and the respondents’ behaviours and perceptions regarding the COVID-19 protection measures that they adopt (Q26) and the perceived risk of a coronavirus infection linked to different daily-life activities (Q21). The questions and possible answers are described in Tables S3 and S4.

**Figure 6:**
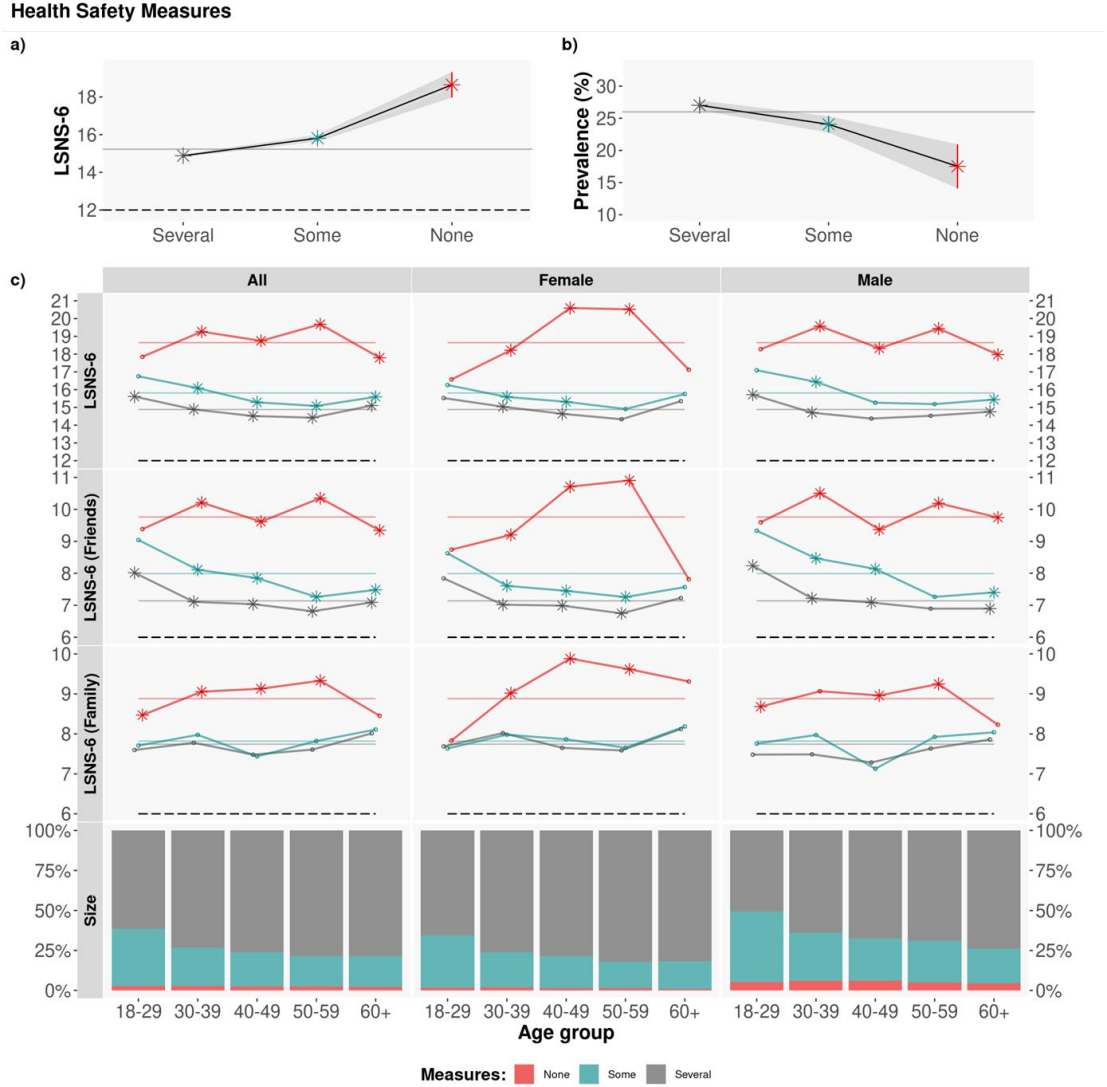
Adopted COVID-19 protection measures. Average LSNS-6 score by age group and gender. The coloured lines correspond to the average Overall/Friend/Family LSNS-6 score per adopted COVID-19 protection measures (Several/Some/None): $\mu _{LSNS-6}$: [14.88, 15.81, 18.64]; $\mu _{LSNS-6\,fam}$: [7.74, 7.82, 8.88]; $\mu _{LSNS-6\,fri}$: [7.14, 7.99, 9.76]. To test the statistical significance of the differences, we applied a Tukey post-hoc test after obtaining a significant difference via an ANOVA test. Asterisks denote statistically significant differences with the rest of groups with $\alpha =0.05$. Those who do not adopt any measures (red lines) have significantly larger LSNS-6 scores than the rest.

As noted in Fig. 6, there is a significant difference in the LSNS-6 scores between those who do not adopt any protection measures (red lines) and the rest of participants (blue and grey lines), with a t-test $p<10^{-16}$ and a difference in means of 3.528. This difference is mainly due to the friends component of the LSNS-6 score: those who do not adopt any COVID-19 protection measures report significantly larger LSNS-6 scores than the rest of respondents, i.e. are significantly less likely to be socially isolated: $\mu _{LSNS-6\,none}-\mu _{LSNS-6\,any} = 3.528 = 2.408~(\hbox {friends}) + 1.120~(\hbox {family})$, with t-test $p<10^{-9}$.

**Figure 7:**
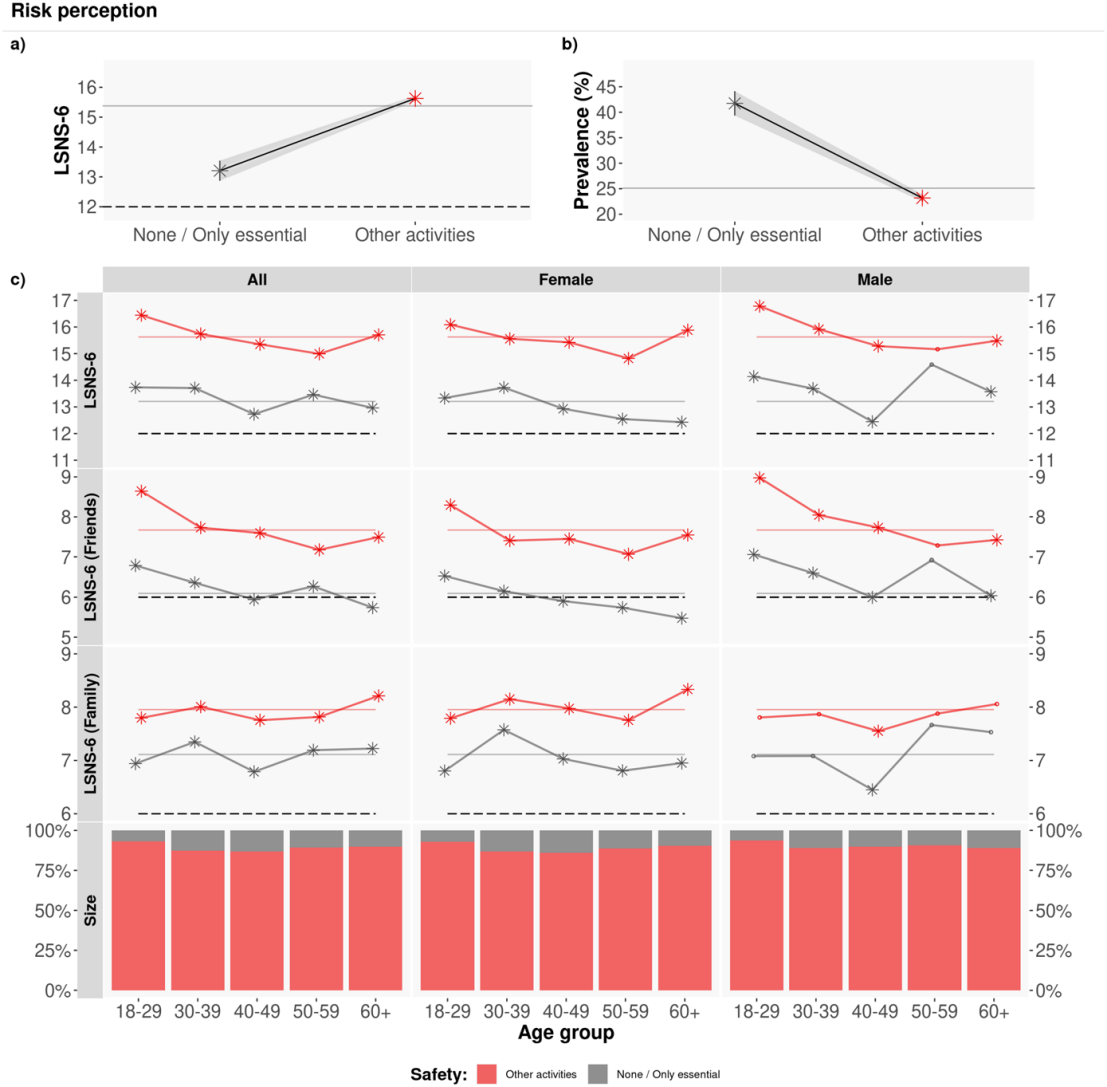
Perception of COVID-19 infection risk for different types of activities. Average LSNS-6 score mean by age group and gender. The coloured lines correspond to the average Overall/Friend/Family LSNS-6 score per adopted COVID-19 protection measures (None/Other): $\mu _{LSNS-6}$: [13.20, 15.63]; $\mu _{LSNS-6\,fam}$: [7.10, 7.95]; $\mu _{LSNS-6\,fri}$: [6.09, 7.68]. The asterisks denote statistically significant differences, with a t-test with $\alpha =0.05$. Those who have a lower perception of risk linked to different daily-life activities (red lines) have significantly larger LSNS-6 scores than the rest of respondents (grey lines).

Next, we focus on the participants’ perception of COVID-19 infection risk associated with activities that are part of our daily lives (Q21) and their levels of social isolation. As depicted in Fig. 7, the LSNS-6 scores of those who report that none or only essential activities can be carried out with low risk of contracting COVID-19 (grey lines) are significantly lower than the LSNS-6 scores of the rest of respondents (red lines). This difference mainly due to the friends component of the LSNS-6 score: $\mu _{LSNS-6\,none-essential}-\mu _{LSNS-6\,other} = 2.436 = 1.586~ (\hbox {friends}) + 0.850~(\hbox {family})$, with t-test * p*-values $<10^{-16}$.

## Discussion

In this paper, we have quantitatively analysed the prevalence of social isolation in Spain during 7 months of the COVID-19 pandemic by means of the LSNS-6 instrument delivered via an online survey with 32,359 answers. We have also studied the relationship between social isolation and the economic and psychological impact of the pandemic on people’s lives. Finally, we have reported the relationship between social isolation, the COVID-19 protection measures and the perception of infection risk associated with common daily activities by our participants. We draw several implications from these results.

### The levels of social isolation have significantly increased during the pandemic

While we do not have data of the prevalence of social isolation as per the LSNS-6 instrument in our sample before the pandemic, the European Quality of Life Survey published in 2017^43^ reports that 78% of European adults and 83.5% of Spanish adults have face-to-face contact at least once a week with their family and friends. According to this report, 75% of European adults and 80% of Spanish adults have telephone or internet contact with their social network at least once a week.

Beyond Spain, a recent German study analysed social isolation data from a sample of 9392 adults (aged 18–79 years old) collected between August 2011 and November 2014. It reports a prevalence of social isolation in their sample of 12.3%^30^. The average LSNS-6 score in their sample was 17.6 ($\hbox {SD} = 5.1$). Those socially isolated had a mean LSNS-6 score of 8.5 ($\hbox {SD} = 2.5$) and the socially integrated individuals of 18.8 ($\hbox {SD} = 4.0$).

In our sample, we find a prevalence of social isolation of 25.98% ($\hbox {SD} = 0.003$), which is almost 14 percentage points larger than that reported in^30^. The average LSNS-6 score in our sample is of 15.23. Respondents who are socially isolated have an average LSNS-6 score of 7.66 and those socially integrated of 17.89. These figures are significantly lower than those reported in the literature.

Our findings are aligned with those of O’Sullivan et al.^35^, who report a prevalence of social isolation of 21% in a study with 14,302 participants from different countries carried out between June and November of 2020. The authors find that 13% of the respondents in their survey experienced a substantial increase in social isolation during the COVID-19 pandemic.

Moreover, we find a statistically significant difference in the prevalence of social isolation among participants who report not adopting any COVID-19 protection measures—and hence do not reduce their social contacts and activities ($prev_{iso} = 17.52\%$)—when compared to participants who report complying with five or more COVID-19 protection measures ($prev_{iso}$ = 27.02%, $\chi ^2$ test $\hbox {p}<10^{-8}$).

This result probably reflects the fact that many of the COVID-19 protection measures are of social nature—such as reducing close contacts, keeping social distance, avoiding gatherings, hugging and kissing—and thus their impact on social isolation is evident.

Hence, we hypothesise that the increase in the social isolation identified in our study is due to the pandemic and the social distancing and confinement measures that have been implemented in Spain to try to contain the spread of the SARS-CoV-2 virus.

### Middle-aged participants are the most socially isolated

Surprisingly, the largest prevalence of social isolation in our sample is among those aged 50–59 years old, with 29.54% of the sample reporting LSNS-6 levels corresponding to social isolation. The LSNS-6 component related to the friends network is the main contributor to this result. This finding might be a consequence of the social distancing and confinement measures adopted by Spain during the period of study. According to our survey, the demographic group of those aged 50-59 years old is among the most compliant with the individual protection measures (washing hands, keeping social distancing, wearing masks, limiting social contacts) while also being a demographic group with a large number of contacts. Hence, the impact of adopting such measures on their friends social network might have been the largest in the sample.

Previous work has obtained similar results in smaller samples and geographies. In a small sample of 214 residents of Wandsworth, a South West London Borough in the United Kingdom^34^, the authors find that middle-aged people reported a weaker social network and more loneliness, anxiety and depression than younger people. Sugaya et al. carry out a study with 11,333 participants from seven prefectures of Japan during the final phase of the state of emergency in May 2020 and report a greater prevalence of social isolation among male, middle-aged (40–64 years) participants^32^.

Interestingly, the U-shaped curve in the LSNS-6 scores that we obtain is similar to the previously reported U-shaped curve of happiness depending on age^44^ where the lowest levels of happiness are reported by those aged 50 years old.

Given the strong correlation between social isolation and mental and physical health, it would be of paramount importance to deploy support programs for individuals particularly in such an age group.

### Economic impact and social isolation are strongly related

Previous work has also found a strong relationship between socioeconomic status and social isolation both before^5,8,37,45–48^ and during the COVID-19 pandemic^30,32,34,49–51^. The general finding that we corroborate here is that the lower the socioeconomic status, the higher the risk for social isolation.

The economic consequences of the COVID-19 pandemic are evident on a global scale, having been responsible for the second largest global recession in history, with more than a third of the global population at the time being placed on lockdown.

However, we are not aware of previous analyses on the relationship between the economic *impact* of the pandemic on an individual and their levels of social isolation. In our data, we find a strong correlation between the economic impact of the pandemic and the LSNS-6 scores of individuals: the lower the economic impact, the larger the LSNS-6 scores and hence the lower the social isolation (see Table 1). The prevalence of social isolation among those who report severe economic impact is significantly larger than the prevalence of the overall sample: $prev_{iso} = 41.39\%$ and $prev_{iso} = 25.98\%$ for those with severe economic impact vs the general population, respectively.

We also observe gender differences, mainly due to the friends component of the LSNS-6 scores: women who have been severely impacted economically because of the pandemic are more likely to be socially isolated ($prev_{iso} = 43.04\%$) than men who also report severe economic impact ($prev_{iso} = 39.78\%$), with average friend LSNS-6 scores that are almost one point smaller than the men’s scores ($\mu _{LSNS-6\,fri-female}$: 6.04 $\mu _{LSNS-6\,fri-male}$: 6.96).

This finding highlights the importance of deploying social programs to support women in vulnerable and economically precarious situations.

### The social support by friends matters

We find that the friends component of the LSNS-6 score is a key determinant of social isolation in our sample across all age groups (see Fig. 1d), more so than the family component. This finding might be due to the fact that families are a fundamental pillar in Spanish culture across all demographic and socioeconomic groups, such that there might not be large differences in the social support provided by families. However, the friends networks vary widely with age, gender, education level and socioeconomic status^52^.

We identify gender differences in the levels of social isolation for those who report severe economic impact due to a much weaker friends social network among women.

There are also significant differences in the friends LSNS-6 scores among those who do not adopt any COVID-19 protection measures (particularly men) and the rest of participants; and those who consider that most activities can be performed with low risk of a SARS-CoV-2 infection and the rest of respondents. In this case, those who do not adopt any COVID-19 protection measures exhibit stronger social support by their friends. Similarly, those who consider that many non-essential activities can be carried out with a low risk of coronavirus infection also have higher LSNS-6 friend scores than the rest of participants.

This finding highlights not only the role that friends play in social isolation—particularly among the youth (see e.g.^53^), but also the influence that having a strong network of friends plays on wanting to socialise even during pandemic times. Alternatively, this result might be explained by the fact that the COVID-19 protection measures are mostly of social nature and hence have an impact on the levels of social isolation of those who comply with them.

### Gender also matters

There are several gender-based differences that can be drawn from our analyses. First, while the average LSNS-6 scores are lower among female ($\mu _{LSNS-6} = 15.18~SD=0.048$) than male ($\mu _{LSNS-6} = 15.29 ~SD=0.074$) respondents, the prevalence of social isolation is larger among male ($prev_{iso} = 26.53\%$) than female ($prev_{iso} = 25.47\%$) participants. This difference, however, is not statistically significant according to a $\chi ^2$ test.

To shed light on this finding, Fig. S2 depicts the distribution of LSNS-6 scores by gender. As can be observed in the Figure, the LSNS-6 scores by female respondents tend to be clustered around the mean of the distribution whereas the upper and lower extremes of the distribution have significantly more male than female respondents. Indeed, we observe a statistically significant difference between the variances of the distributions with a much larger support of answers by male participants in the tails of the distribution when compared to the answers by female respondents (see Tables S1 and S2).

Second, women with severe economic impact are significantly at higher risk of social isolation than men who have also suffered severe economic impact, mostly due to having a weaker support by their friends. Interestingly, this difference is not evident among those with psychological impact due to the pandemic.

### The adoption of COVID-19 protection measures is related with social isolation

When we divide our sample according to the COVID-19 protection measures adopted by the participants in our study, significant differences emerge in the respondents’ levels of social isolation: those who report not adopting any measures have significantly larger LSNS-6 scores than those who adopt some or all of the possible COVID-19 protection measures (None/Some/Several: $\mu _{LSNS-6}$: [18.65, 15.81, 14.88], ANOVA and Tukey tests $p<10^{-16}$.

This finding might be explained by the role that the friends network plays in influencing human behaviour. Many of the COVID-19 protection measures are meant to limit our social interactions. Those with strong friends networks are therefore more impacted by the adoption of such measures and hence could be less likely to adopt them. As previously reported, the difference in the LSNS-6 scores between those who do not adopt any measures and the rest of participants is of 3.524 points, mostly due to the friends component of the LSNS-6 score.

Alternatively, this result might be explained by the fact that the COVID-19 protection measures are mostly of social nature and hence have an impact on the levels of social isolation of those who comply with such measures.

### Higher perception of COVID-19 infection risk is related to social isolation

Similarly, those who report that many daily activities can be performed with a low risk of a coronavirus infection are significantly less likely to be socially isolated than those who have a higher perception of infection risk: $prev_{iso}$ = 23.13% vs. $prev_{iso}$ = 41.73% ($\chi ^2$ test with $p<10^{-16}$). Again, the LSNS-6 component that contributes the most to this difference in LSNS-6 values (13.20 vs. 15.63) is the friends component: of the 2.44 points in the difference of the average LSNS-6 scores, 1.59 points are due to the friends component and 0.85 points due to the family component, with a t-test $p<10^{-16}$, and with a larger difference between female than male respondents.

Our results might be explained by several factors. First, previous work has found that the larger the social support, the lower the fear towards COVID-19 in a sample of healthcare workers in Jordan^54^. Second, in the context of the coronavirus pandemic, we tend to lower our perception of infection risk when carrying out activities that involve our friends and/or relatives^55^: “our evaluation of risk versus safety is inextricably tied to our group memberships”.

Several of our findings highlight the crucial role that the friends support network plays in the levels of social isolation in Spain.

## Materials and methods

### Data collection and processing

In this paper, we analyse a subset of the answers to the COVID19ImpactSurvey, an extensive, anonymous, online citizen survey about COVID-19^40^. Launched on March, 28th 2020, in Spain, the survey has since then collected over 700,000 anonymous answers from 11 countries (mostly Spain, Italy, Germany and Brazil), and it is available online at https://covid19impactsurvey.org. Participants must declare being 18 years or older to be able to fill the survey. All research was performed in accordance with relevant guidelines/regulations. Informed consent was obtained from all users and the data was collected in de-identified form.

We filter entries with inconsistent answers (5.19% of answers), which are entries where the respondents provided contradictory answers, such as reporting being a pregnant and aged 60+; being pregnant and male, etc. We also do not consider entries that appear to have been answered too fast (< 120 s) or too slow (> 36000 s), which correspond to 3.34% of answers. Note that the average completion time of the survey in our sample is 509 seconds. Finally, entries not providing age or male/female sex information (0.81%) are also excluded from the analysis. In total, we discard 9.34% of the answers.

The specific answers to the survey that we analyse in this paper collect information about the participants’ demographic and household situation (Q1–Q5); their LSNS-6 scores (Q11–Q16); the economic impact of the pandemic in their lives (Q20, Q22); their perception of COVID-19 infection risk associated with different activities (Q21); the individual protection measures that they adopt to protect themselves against COVID-19 infections (Q26); the psychological impact of the pandemic in their households (Q31). All the questions to the survey are included in Table S3.

We analyse the data from Spain for the time period between June 6th, 2021 and the 16th of December, 2021. During this period, Spain had returned to the “new normality”: the only COVID-19 protection measure implemented in the country was the requirement to wear face masks in indoor public spaces. Figure S3 depicts the daily number of new COVID-19 cases and deaths in Spain. The period of study includes a small wave of infections that took place in the summer of 2021. Survey answers were collected from volunteer respondents who learned about the survey via social media channels, word-of-mouth, universities and news organisations. We used Facebook and Instagram ads as an additional channel to recruit volunteers. This approach gave us a straightforward method to obtain a broad sample of users across different socioeconomic and demographic groups. We did not use any targeting feature except for gender, where we used separate budgets to balance the numbers of male and female respondents. The cost-per-successful-response was €0.24 and €0.11 for men and women respectively. Both men and women had similar completion rates (54%) once they began answering the survey.

The age and gender distribution of our sample is different from the population distribution in Spain. Thus, we follow the methodology described in Oliver et al.^40^ and re-weight the answers such that the resulting gender and age distributions match the official statistics of each of the seven Nielsen geographical regions in Spain, depicted in Fig. S1, plus the metropolitan areas of Madrid and Barcelona. We compute the weights according to the official data reported by the Spanish National Institute of Statistics (INE) for 2020.

To further validate our methodology, in April 2020 we commissioned an IPSOS.digital FastFacts panel of a cohort of 1000 representative general population respondents aged 18–65 in Spain. This validation was done after we started using Facebook ads, but before the time period covered by this paper. The results of the IPSOS panel were within the margin of error of our survey results for the same time period and ages.

#### Ethical approval

All methods described in this manuscript were carried out in accordance with relevant guidelines and regulations. The survey instrument was approved by the Ethical Committee of the University Miguel Hernandez, which is called the Office for Responsible Research (OIR), in the context of previous research^40,41^. Informed consent was obtained from all subjects who had to confirm being at least 18 years old to be able to participate in the survey. The participation in the survey was voluntary and all the collected data was anonymous and confidential. Furthermore, the research complied with the ethical standards of the 1964 Declaration of Helsinki and its later amendments.

### Measures

All the measures used in our analysis are described in Tables S3 and S4.

#### Social isolation

We measure social isolation using the LSNS-6 instrument included in questions Q11–Q16 in the survey. The LSNS-6 is a quantitative measure of an individual’s social network size that assesses both the number and frequency of contacts with friends and family and the social support received by them. It consists of six items: three questions ask about the frequency of meeting relatives, how many relatives the respondent feels close enough to ask for help and with how many relatives the respondent can talk about private matters; the remaining three questions are identical but concerning friends rather than relatives. Each of the LSNS-6 items is scored on a scale from 0 to 5 and they are all equally weighted. Thus, the total score ranges between 0 and 30. The higher the LSNS-6 scores, the lower the levels of social isolation. Social isolation is given by a score below 12. This corresponds to having less than 2 individuals to reach out to in each of the areas probed by the survey (i.e. family or friends). The LSNS-6 has good psychometric properties^13^.

#### Socio-demographic and household characteristics

Demographic variables selected for analysis were: (1) gender, where respondents were asked to self-report their gender as male, female or other. In this study, we only consider the answers of those who responded as male or female, which represent 99.33% of the answers (0.3% of answers selected “other” as their sex and 0.37% of answers did not provide sex information); (2) age, coded in the following bins: 18–29; 30–39; 40–49; 50–59; 60+; (3) zip code of their residence; and (4) number of members in the household, coded as: 1, 2, 3, 4+.

### Economic impact

The self-reported economic impact of the pandemic was assessed in Q20 and Q22 of the survey.

Question number 20 asks about the type of economic impact that the pandemic had on the respondents’ lives. Based on the answers provided to Q20, we coded the severity of the economic impact in four levels: [None, Mild, Severe and Positive], as described in Table S3.

Question number 22 focuses on whether participants receive any kind of economic assistance from the government.

Based on the answers provided to Q22, we coded the reliance on government-based economic assistance in two levels: [None, Any], as described in Table S3.

#### Psychological impact

The self-reported psychological impact of the pandemic was assessed in Q31 of the survey.

Based on the answers provided to Q31, we code the severity of the psychological impact in three levels: [None, Mild and Severe], as described in Table S3.

#### COVID-19 protection measures

The individual protection measures adopted by participants to limit their exposure to the SARS-CoV-2 virus were captured by Q26.

We code the possible answers into three groups: [All, Some, None], depending on whether respondents report adopting all, none or several of the measures, as described in Table S3.

#### Perception of COVID-19 infection risk

Question 21 asked participants about their perception of risk of a COVID-19 infection associated with different daily-life activities.

We code the possible answers into three groups: [None, Only essential activities and Other non-essential activities], as depicted in Table S3.

### Statistical analysis

The prevalence of social isolation is computed in percent with 95% confidence intervals (CIs) from the overall sample. We stratify social isolation by gender and age. We perform $\chi ^2$ tests to determine the existence of statistically significant differences in the prevalence of social isolation among each of the stratified groups. We also use multi-variable logistic regression analysis to test the associations between social isolation and age, gender, number of members of the household, economic and psychological impact of the pandemic, individual COVID-19 protection measures adopted and risk perceptions of coronavirus infection associated with different daily activities. We use a significance level $\alpha =0.05$ (two-tailed) in all the analyses.

All statistical analyses, data preprocessing and figures were performed and plotted using the R software^56^ package, version 4.1.2 (2021-11-01).

### Limitations

Collecting a large sample of answers via an online survey does not come without limitations. Even if the data size is large and the confidence intervals are small, errors might compound and under- or over-estimate the results^57^. Moreover, even if we weighted^59^ the data to mitigate gender and age survey’s biases in each Nielsen region, and we deployed gender-balanced Facebook advertisement campaigns, the data is non-random and biases might still be present. For example, there are self-selection and sampling biases^59^ as the survey is filled out by volunteers who have learned about the survey via social media, WhatsApp, newspapers’ articles or Facebook ads, and who need to have access to a computing device (smartphone, tablet, PC) with an Internet connection. The survey might also be biased towards people who are more likely to fill-in surveys, such as young and highly educated people. Respondents must be adults—at least 18 years old. Hence, students are only partially covered. We acknowledge all these biases challenge the estimation of inferential statistics, and might lead to overconfidence in incorrect inferences due to the collected large data sample and small confidence intervals^57^. We also acknowledge that there might be a recall bias as respondents were asked about their perceptions and behaviour in the last seven days (e.g. the number of close contacts in the last week). However, we have taken several measures (re-weighting, contrasting with two polls, comparing with relevant literature) to minimise such biases. Previous work has found that citizen (online) surveys are particularly valuable tools in situations of data scarcity where informed and timely decisions are needed^40^. Online surveys also allow monitoring people’s perceptions and behaviours, enabling the design of more effective public policies and better education and communication with the public. Thus, we are confident about the value and validity of the results of our analyses.

## Supplementary Information

Supplementary Information.

## Data Availability

All data generated or analysed during this study are included in this published article [and its supplementary information files].

## Code availability

The code used to analyse the data will be placed in a publicly available Github server.
