# Supplementary material for: Social isolation during the COVID-19 pandemic in Spain: a population study
Source: Sci Rep. 2022 Jul 22;12:12543. doi: 10.1038/s41598-022-16628-y (PMC9306252; doi:10.1038/s41598-022-16628-y)
Supplement: Supplementary file 1 — Supplementary Information. [file 41598_2022_16628_MOESM1_ESM.pdf]

Supplementary Information: Social isolation during the COVID-19 pandemic in Spain: a population study

Correspondence and request for materials should be addressed to M.M-G.

Survey questions Table S3 depicts the questions whose answers have been analysed in this paper. The survey has a total of 31 questions. However, there are a few conditional questions such that the number of the questions is that a person might answer could be more than 31. There are gaps in the numbering since during the lifetime of the survey questions regarding the lockdown behaviour were removed as they were no longer relevant. Table S4 depicts the dependent and independent variables used in this study.

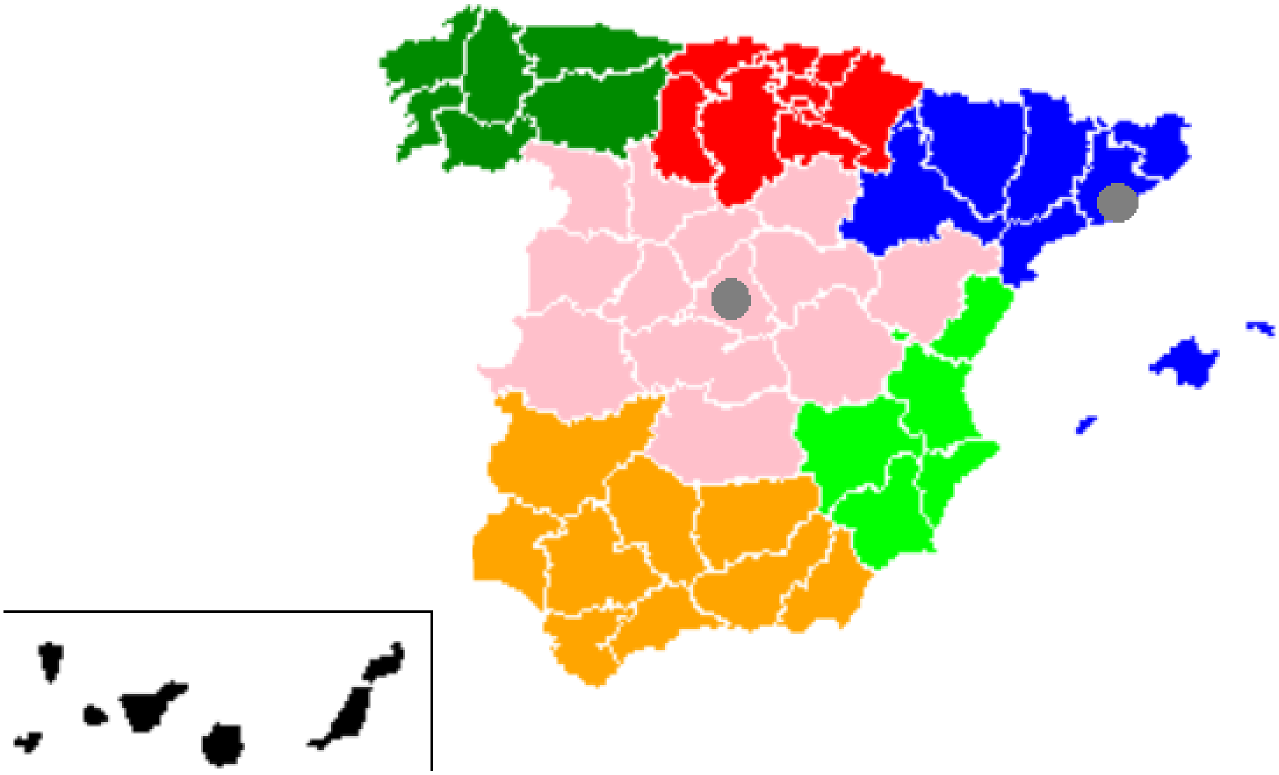

Figure S1. The 7 Nielsen areas plus the metropolitan areas of Madrid and Barcelona, used to reweigh the data.

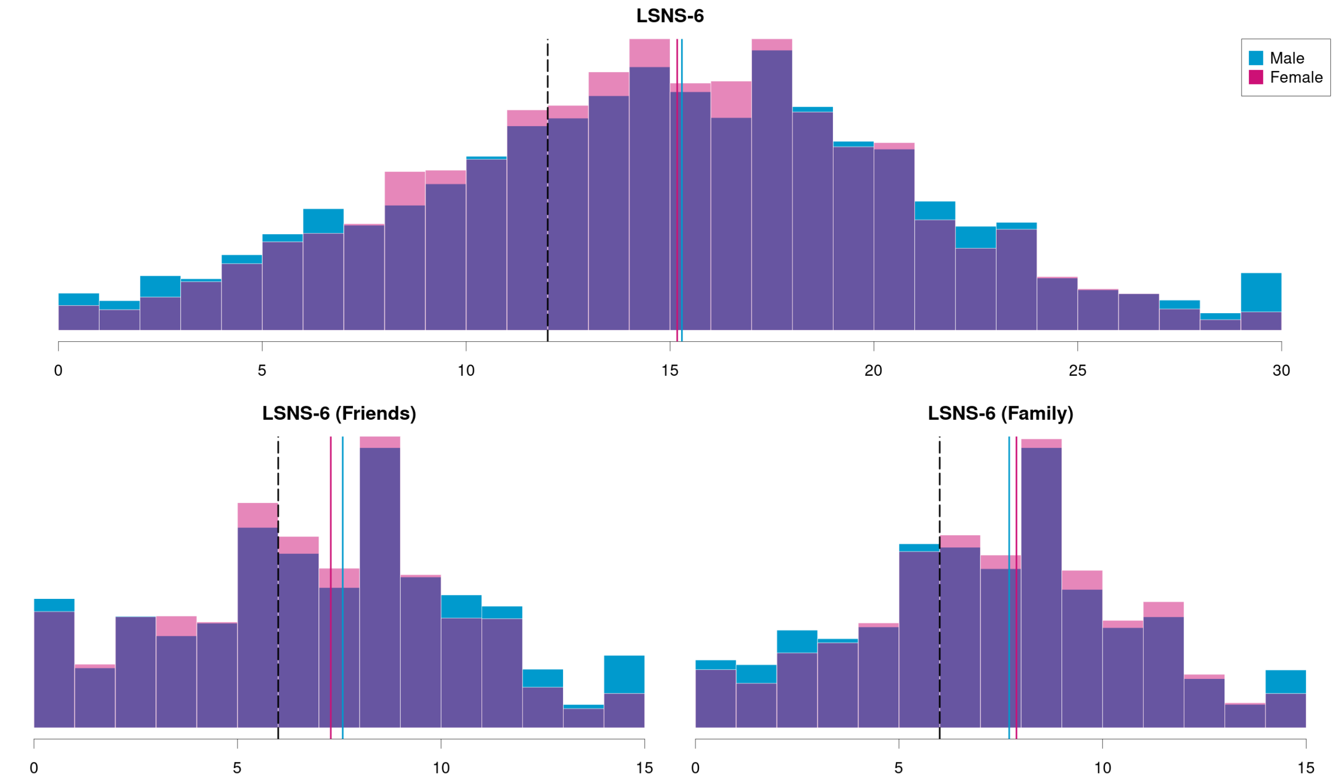

Figure S2. Distribution of LSNS-6 scores by gender: male (blue) and female (pink) respondents. The vertical lines show the average LSNS-6 score by gender, and the dashed vertical lines show the threshold for social isolation: LSNS-6 score <12

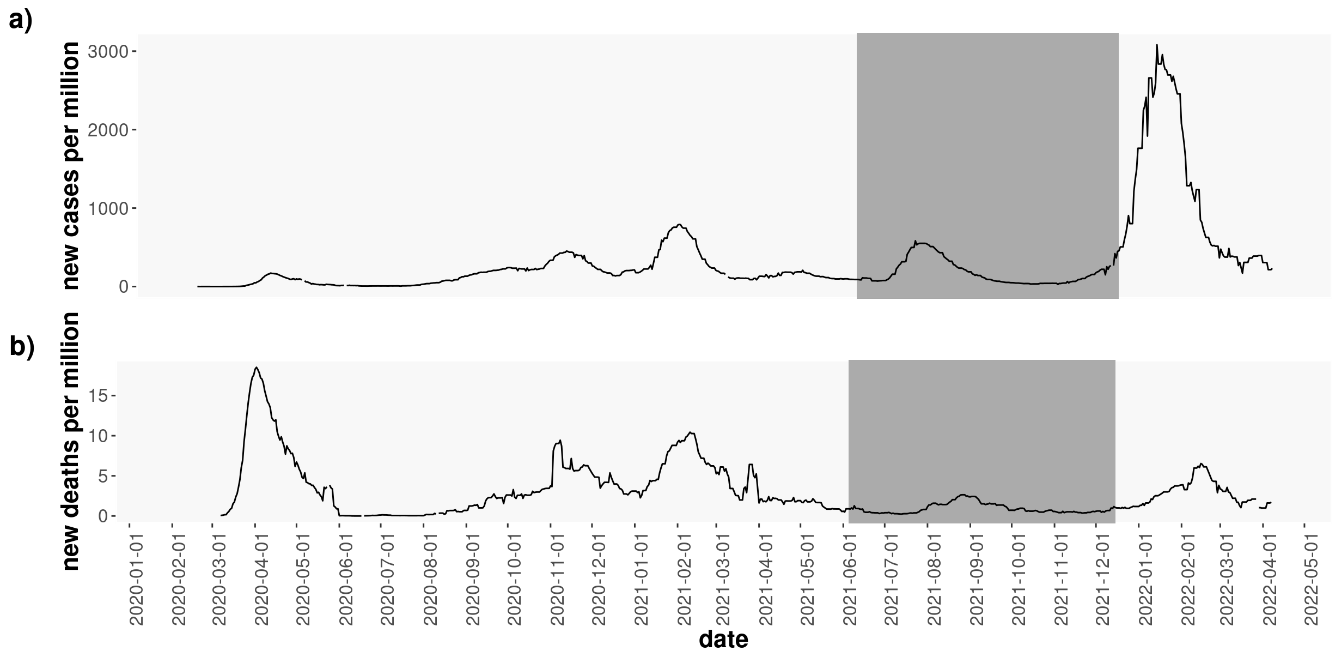

Figure S3. Evolution of the COVID-19 pandemic in Spain: a) New cases per million, and b) new deaths per million. The gray area marks the time period of study.

	Point estimation	Normal Fitt
Female		
μ	15.18	15.13
σ	5.67	5.57
Male		
μ	15.29	15.36
σ	6.17	6.13

Table S1. Estimations of the parameters of the above distributions: There is no statistical differences between the means according to a t-test, yet we observe a statistically significant difference between the variances of both distributions with a Levene test p$< 10^{16}$

LSNS-6 score limits	$\leq 3 and \geq 28$	$\leq 4 and \geq 27$
Female	3.10 %	5.16 %
Male	5.66 %	16.30 %

Table S2. Percentage of participants in the tails of the distributions of LSNS-6 scores by gender. Note the large percentage of male respondents in the tails of the distribution when compared to female respondents.

Table S3. COVID19ImpactSurvey questions analysed in our study.

**Question**	**Possible answers**
**Demographic and Household information**	
Q1. What is your age range?	[18-20; 21-29; 30-39; 40-49; 50-59; 60-69; 70-79; 80+]
Q2. What is your gender?	[Male; female; another gender]
Q3. Postal code/Zip code	Text entry
Q4. Type of home	[Single family house; apartment/flat; shared apartment/flat; other shared accommodation; other]
Q5. Number of people in the home (including you)	[1; 2; 3; 4; 5+]
**Social Behaviour**	
Q9. In the last seven days, approximately how many different people that live outside your home have you had close contact with? (meaning more than 15 minutes and a distance closer than 2 meters)	[No one; 1-2; 3-4; 5-9; 10-19; 20-49; 50+]
**LSNS-6**	
Q11. How many relatives do you see or hear from at least once a month? (considering the people to whom you are related by birth, marriage, adoption, etc.)	[No one; 1;2;3-4;5-8;9+]
Q12. How many relatives do you feel at ease with that you can talk about private matters?	[No one; 1;2;3-4;5-8;9+]
Q13. How many relatives do you feel close to such that you could call on them for help?	[No one; 1;2;3-4;5-8;9+]
Q14. How many of your friends do you see or hear from at least once a month? (considering all of your friends including those who live in your neighborhood)	[No one; 1;2;3-4;5-8;9+]
Q15. How many friends do you feel at ease with that you can talk about private matters?	[No one; 1;2;3-4;5-8;9+]

Q16. How many friends do you feel close to such that you could call on them for help?	[No one; 1;2;3-4;5-8;9+]
Q19. Do you believe that the measures the government has taken are enough to contain the spread of coronavirus?	[No, but should be stricter; Yes, are about right; Yes, but are too strict; Prefer not to respond; I do not know]
**Economic Impact**	
Q20. What kind of economic impact has the coronavirus had on you? (check all that apply)	[I lost my job; I lost my savings; I cannot pay my rent or mortgage anymore; I cannot afford to buy food; I have lost most or all of my income; My business is in danger of bankruptcy; My employer is in danger of bankruptcy; My employer has reduced by working hours due to lack of demand; I have a new job or business opportunity; I have significantly increased my savings or reduced my debt because I am spending less; None of the above]
Q22. In the last month, have you received any of the following economic assistance from your government? (check all that apply)	[Retirement (pension or social security); Unemployment benefit (temporary); Unemployment benefit (I lost my job); Universal Basic Income; Using the moratorium to reduce my taxes, mortgage, rent or other debt payments; One-time bonus to cover COVID expenses or stimulate the economy; Social housing or subsidised rent; Assistance for my business that I have to pay back (like a loan); Assistance for my business that I do not have to pay back; Disability benefits; Other type of welfare (due to poverty, illness or other reason); None of the above]
**Perception of infection risk**	
Q21. Which of the following activities do you think can be done with a low risk of coronavirus infection? (check all that apply)	[Practising individual sports; Having friends visit you at home; Attending religious services with limited seating; Attending school like in some European countries; Going to small businesses with appointment (hairdresser, etc); Going to small shops while maintaining a safe distance; Having drinks at a bar on an open terrace with a group of people; Going to restaurants with limited seating; Receiving treatment at a hospital; Taking public transportation with space between seating; Going to the beach; Travelling by air; None of the above]
**COVID-19 infection protection measures**	
Q26. Do you take any of the following measures to prevent the transmission of the coronavirus? (check all that apply)	[I wear a mask as much as possible; I avoid crowded situations; I don't shake hands, give hugs or kisses to anyone who live outside my home; I regularly disinfect/wash my hands; I keep my physical distance of at least 1.5 meters (6 feet) from others; I limit the number of people that I am in close contact with When indoors; I make sure there is good ventilation; I have installed my government's contact tracing app on my phone; I would be willing to get vaccinated immediately when the coronavirus vaccine is available; None of the above]
**Psychological Impact**	
Q31. Have you noticed a significant increase in your home in any of the following areas that you consider damaging? (check all that apply)	[High level of anxiety; High level of stress; High level of loneliness; High level of sadness; Loud arguments or fights with other members of the home; Excessive consumption of alcohol; Excessive consumption of drugs (prescription or other); Excessive use of technology by adults (tablet, phone, TV); Excessive use of technology by children (tablet, phone, TV); I have not noticed a harmful increase in these areas; I prefer not to answer]

Table S4. Independent and dependent variables used in our study.

Variable/Values	Explanation
Socially Isolated	
1	LSNS-6 score < 12
0	LSNS-6 score ≥ 12
Age (Q1)	
18-29	18 to 29 years old
30-39	30 to 39 years old
40-49	40 to 49 years old
50-59	50 to 59 years old
60+	60 or more years old
Gender (Q2)	
Male	Male respondents
Female	Female respondents
Number of people in the home (Q5)	
"1"	People who answered "1"
"2"	People who answered "2"
"3"	People who answered "3"
"4+"	People who answered "4+"
Close contact in the last 7 days (Q9)	
No one	People who answered "No one"
1-2	People who answered "1-2"
3-4	People who answered "3-4"
5-9	People who answered "5-9"
10-19	People who answered "10-19"
20-49	People who answered "20-49"
50+	People who answered "50+"
Economic Impact (Q20)	
None	No economic impact is reported
Mild	I lost my job;I lost part or all of my savings; I have lost most or all of my income; My business is in danger of bankruptcy; My employer is at risk of bankruptcy; My employer has reduced my hours due to lack of demand
Severe	I can't afford to buy food; I can't pay my rent or mortgage anymore
Positive	I have a new job or business opportunity; I have significantly increased my savings
Activities with perceived low risk of COVID-19 infection (Q21)	
Classification of activities	
**Essential:**	Receiving treatment at a hospital; Attending school like in some European countries; Going to small shops while maintaining a safe distance; Taking public transportation with space between seating; Going to small businesses with appointment (hairdresser, etc);
**Non-essential:**	Going to restaurants with limited seating; Going to the beach; Attending religious services with limited seating; Having drinks at a bar on an open terrace with a group of people; Practising individual sports; Travelling by air; Having friends visit you at home;
Grouping	
None/Only essential	No activity, or only those that are essential, can be safely reopened: People who answered Yes to open at least one essential and "No" to all other activities
Other activities	People who answered Yes to open at least one essential activity, and answered Yes to at least one of the other activities.

Government eco. assistance (Q22)	
None	People who do not receive any government support
Any	People who receive at least one of the following: Assistance for my business that I have to pay back (like a loan); Assistance for my business that I do not have to pay back; Disability benefits; Assistance cash, Assistance loan, One-time bonus to cover COVID expenses or stimulate the economy; Using the moratorium to reduce my taxes, mortgage, rent or other debt payments; Social housing or subsidised rent; Unemployment benefit (I lost my job); Unemployment benefit (temporary); Universal Basic Income
COVID-19 Protection Measures (Q26)	
None	People who are not taking any health safety measures None of the above
Some	People who answered "Yes" to at most 4 health safety measures
Several	People who answered "Yes" to 5 or more safety measures
Psychological Impact (Q31)	
None	No psychological impact is reported
Mild	Excessive use of technology by adults (tablet, phone, TV); Excessive use of technology by children (tablet, phone, TV)
Severe	High levels of anxiety;High levels of stress; High levels of loneliness; High levels of sadness; Loud arguments or fights with other members of the home; Excessive consumption of alcohol; Excessive consumption of drugs (prescription or other)

Table S5. Distribution of answers per social isolation values.

Variable	Responses		LSNS-6		Prevalence	
	raw	reweigh	raw	reweigh	raw	reweigh
Socially Isolated						
1	25.54 %	25.98%	7.81	7.66	-	-
0	74.46 %	74.02%	17.73	17.89	-	-
Age (Q1)						
18-29	21.58%	15.03%	15.95	16.14	19.90%	19.68%
30-39	17.17%	15.38%	15.25	15.37	24.03%	23.72%
40-49	20.37%	20.05%	14.76	14.84	29.14%	29.48%
50-59	19.97%	18.15%	14.62	14.73	29.78%	29.55%
60+	20.91%	31.40%	15.36	15.27	25.03%	25.82%
Gender (Q2)						
Male	28.22%	48.51%	15.36	15.29	25.94%	26.53%
Female	71.78%	51.49%	15.13	15.18	25.38%	25.47%
Number of people in the home (Q5)						
1	10.69%	13.13%	14.59	14.38	28.54%	30.77%
2	33.18%	36.53%	15.19	15.29	25.11%	25.25%
3	26.57%	24.60%	15.04	15.09	26.01%	26.22%
4+	29.56%	25.74%	15.57	15.76	24.31%	24.03%
Close contact in the last 7 days (Q9)						
No one	9.50%	9.24%	12.31	12.10	45.02%	46.00%
1-2	16.41%	15.79%	12.49	12.25	42.44%	43.98%
3-4	20.68%	20.07%	14.28	14.20	28.09%	28.93%
5-9	23.19%	22.73%	16.07	16.06	17.63%	18.52%
10-19	16.64%	17.38%	17.32	17.30	14.29%	15.21%
20-49	7.77%	8.18%	18.02	18.07	12.26%	11.95%

50+	5.81%	6.60%	17.51	18.08	18.70%	17.28%
Economic Impact (Q20)						
None	58.11%	60.07%	15.42	15.40	24.19%	24.79%
Mild	22.62%	20.98%	14.56	14.71	29.48%	29.30%
Severe	3.85%	3.68%	12.89	13.09	42.30%	41.39%
Positive	15.41%	15.27%	15.89	15.84	20.51%	22.17%
Activities with perceived low risk of COVID-19 infection (Q21)						
Only essential	10.52%	10.28%	13.21	13.20	41.56%	41.73%
Other activities	89.48%	89.72%	15.60	15.63	22.63%	23.13%
Government eco. assistance (Q22)						
None	84.59%	84.13%	15.33	15.34	24.76%	25.32%
Any	15.41%	15.87%	14.38	14.50	30.65%	31.01%
COVID-19 Protection Measures (Q26)						
None	2.5%	3.17%	18.49	18.64	16.52%	17.53%
Some	24.03%	24.49%	15.78	15.81	23.20%	24.06%
All	73.48%	72.34%	14.89	14.88	26.62%	27.02%
Psychological Impact (Q31)						
None	30.6%	34.73%	15.97	16.01	22.42%	23.08%
Mild	13.6%	13.82%	15.84	15.85	20.60%	20.48%
Severe	55.8%	51.51%	14.63	14.54	28.26%	29.28%
